# Lived experience inclusion in psychology education: a survey of Australian tertiary institutions

**DOI:** 10.1080/00049530.2025.2511882

**Published:** 2025-06-02

**Authors:** Kim L. Johnston, Judith Gullifer

**Affiliations:** School of Psychological Sciences, Monash University, Clayton, Australia

**Keywords:** Lived experience, living experience, psychology education, mental health education, psychosocial safety

## Abstract

**Objective:**

To conduct a preliminary survey of staff involved in teaching accredited psychology units at Australian tertiary institutions about their inclusion of lived experience in education.

**Method:**

Academics were informed about the study by Heads of School/Directors of Education. Thirty-two educators across undergraduate and postgraduate psychology courses completed an online survey. Content analysis was used to identify recurring themes and patterns in the data.

**Results:**

Over 50% of the respondents were using their own or others’ lived experience in their curriculum, with the primary reason being to enrich learning. The main barriers reported were resource constraints, perceived relevance, and work-safety concerns. Key enablers were identified as resourcing, leadership support, and increasing acceptability of lived experience. Almost two thirds of respondents self-identified as having personal lived experience.

**Conclusions:**

This study provides an initial snapshot of the current state of lived experience inclusion in Australian psychology tertiary education. The findings are of importance for our discipline to maintain consistency with other disciplines and ensure we are preparing graduates to effectively contribute to a workforce which values consumer and community expertise.

## KEY POINTS

**What is already known about this topic**:

1. Involvement of people with lived experience has expanded considerably in recent years across education, research, policy, and practice.
2. Many health profession education courses now include lived experience content and expertise, however not much is known about inclusion in psychology education.
3. Competency standards require psychology graduates to work with respect and sensitivity towards people with diverse experiences and perspectives.

**What this topic adds**:

1. This is the first known survey of educators at tertiary institutions offering accredited courses in psychology in Australia about the inclusion of lived experience in teaching/curriculum.
2. Over one-half of survey respondents are using their own or others’ lived experience in their psychology teaching/curriculum, while others would like to but have not yet had the opportunity, seeing it as a way to enrich learning.
3. Barriers to the inclusion of lived experience in Australian tertiary psychology courses included resource constraints such as time and budget, as well as the perceived relevance of lived experience to psychology teaching/curriculum.

Lived (and/or living) experience in the context of psychology generally refers to the knowledge and insight someone has obtained through their personal experiences of mental ill-health, service use, or recovery. It also encompasses related experiences that may impact mental health and wellbeing such as disability, circumstance, marginalisation, discrimination, and community identity. The unique insights gained through this personal experience can occur in relation to an individual’s experience or as a family member/supporter of an individual. The World Health Organization (2022) advocates for meaningful engagement and power sharing with people who have lived experience. Consistent with international developments, in Australia the recognition and prioritisation of this expertise has increased substantially across government, research, training, and service provision sectors (e.g., National Lived Experience (Peer) Workforce Development Guidelines, Byrne et al., 2021; National Mental Health and Suicide Prevention Plan, Commonwealth of Australia, 2021).

There has also been a strong movement towards inclusion of lived experience in the tertiary education of health and social care professions (in particular psychiatry, medicine, nursing, and social work). Research ethics boards and grant applications require inclusion of people with lived experience on investigator teams, and accreditation requirements of some disciplines mandate service user involvement in the curriculum (e.g., occupational therapy; Scanlan et al., 2022). In the context of tertiary education, the literature most often describes the involvement of people with lived experience in teaching through guest lectures, sharing narratives about experiences, or to support practical skill development. More recent is increasing involvement of lived experience in curriculum development, assessment, and governance (Fossey et al., 2024). Some institutions have employed designated lived experience educators (see, for example, a Curtin University initiative outlined in Mahboub et al., 2022), while others have pioneered mental health education co-design frameworks, such as the work of Brand et al. (2021).

Evidence demonstrates that including lived experience in the curriculum promotes deep learning through connecting theory with its real-world expression (Goodhew et al., 2022). Further, it has been shown that interaction with people who have lived experience may help reduce stigma through correcting student misconceptions, positively affecting student attitudes, and expanding understanding of recovery (Horgan et al., 2018; Whitelaw et al., 2022). Interaction with people who have gained expertise through lived experience builds communication and self-reflection skills, supporting the development of students’ capacity to work with a diverse range of individuals and scaffolding them to the competencies required for the workforce (Happell & Bennetts, 2016). On a personal level, the inclusion of lived experience in tertiary education can support psychosocial safety and self-acceptance for students and staff who themselves may have lived experience, and with appropriate structures in place has also been shown to improve the wellbeing of collaborators (Byrne, 2017; Haywood et al., 2023; Woof et al., 2021).

## Consumer and community involvement in health professions education

Within the education of other health professions, research has been conducted to investigate the barriers and enablers of including lived experience in mental health related curriculum (e.g., Fossey et al., 2024; Gee et al., 2016; Happell et al., 2015; Scholz et al., 2018). The challenges of adequate funding and bureaucratic structures within universities have been recognised as key barriers to the involvement of people with lived experience (Fossey et al., 2024). There have also been concerns raised about the reliability and vulnerability of people with lived experience, meeting needs within the university environment, and the psychosocial safety of students and lived experience collaborators (Happell et al., 2015). Some authors highlight difficulties among academic staff who prioritise objective, scientific, and professional knowledge in recognising the expertise that those with personal experience can offer (Gee et al., 2016; Scholz et al., 2018). In addition, advancing the views of one person has been seen by some academics as a limitation and not representative of the collective experience (Happell et al., 2020).

Enablers of the inclusion of people with lived experience often relate to modifying the characteristics of the educational institution. Happell et al. (2022) acknowledge the importance of funding, having appropriate structures in the institution, and momentum in the organisation to facilitate inclusion of lived experience. These enablers also allow for extension to more embedded approaches, such as through the use of an “allies” programme: having a person with lived experience partner with an academic staff member to support and advocate for purposeful participation (Dorozenko et al., 2016). Preparation of people with lived experience, through explaining the practices and language of the higher education setting, are important considerations for successful inclusion (see, for example, the work of Molloy et al., 2023).

## Lived experience inclusion in psychology

Published papers in psychology on the lived experience of practitioners, researchers, and academics are limited but increasing (e.g., Bhattacharya, 2022; Devendorf et al., 2023; Varghese & Boyd, 2022; Victor, Devendorf, et al., 2022; Victor, Schleider, et al., 2022). For example, the American Psychological Association’s publication *Psychological Services* had a special edition in 2022 on “prosumers”: mental health clinicians with their own lived experience of mental ill-health and recovery. In a North American survey of faculty, graduate students, and affiliates of doctoral programmes in counselling, clinical, and school psychology, Devendorf et al. (2023) found that over half of the respondents conducted self-relevant research, with this practice being more common among those from minority backgrounds. Psychologists with lived experience are recognised as having acute insights across research, education, and clinical work, which can be crucial for encouraging mental health recovery (Varghese & Boyd, 2022; Victor, Schleider, et al., 2022).

Given this knowledge, and the burgeoning inclusion of lived experience across tertiary health profession education, it could be presumed that in order to prepare psychology students for the workforce, psychology courses would have an increasing focus on lived experience-related interprofessional content or inclusion of people with lived experience. However, studies showcasing lived experience inclusion in tertiary education of psychology students are rare. Most research focuses on postgraduate clinical training, and is often specific to the United Kingdom where it is part of the British Society of Psychology accreditation of postgraduate clinical training to include service users (Clarke & Holttum, 2013). This discrepancy with other health professions education likely relates to the complexity of psychology education as being education in the discipline as well as the profession, with professional training only commencing at a postgraduate level.

Nevertheless, the absence of lived experience inclusion across undergraduate to postgraduate tertiary psychology education seems misaligned with broader policy and practice in Australia, where consumer and community voice are embedded as fundamental across research and service delivery more broadly. Psychology Board of Australia requirements for graduates who can “demonstrate a health equity and human rights approach” and “ability to reflect on and learn from people’s unique experiences” also underscores the potential value of including lived experience expertise across curriculum (Psychology Board of Australia, 2024). The aim therefore of the current study was to conduct a preliminary survey of educators at Australian tertiary institutions providing accredited psychology programmes to explore their use of lived experience in teaching/curriculum, and secondary to this, capture perspectives of educators who self-identify as having lived, in addition to professional, expertise. Our exploratory research questions were:

1. How is lived experience being used in accredited tertiary psychology courses in Australia?
2. What are the reasons psychology educators choose to include lived experience in the curriculum?
3. What do psychology educators perceive to be barriers for including lived experience in the curriculum?
4. What do psychology educators identify as the enablers for including lived experience in the curriculum?

## Materials and methods

### Participants

In this preliminary study, a total of 32 respondents returned a completed survey (62.5% women, 31.3% men, with one respondent identifying as non-binary/gender diverse and one preferring not to respond). Survey responses primarily came from academics in Queensland (37.5%) and Victoria (25.0%), with other respondents teaching at institutions in Australian Capital Territory, New South Wales, and South Australia. Respondents taught in undergraduate (*n* = 12), postgraduate (*n* = 8), or across courses (*n* = 12), and academic level ranged from Professor (*n* = 10) to Lecturer (*n* = 6) with most respondents being in a Teaching and Research position (68.8%). A third of the respondents had worked as practitioners (*n* = 11) and 22 respondents (71.0%) identified as having their own psychology-related lived experiences as an individual or carer/significant other. Further description of the sample can be found in Table 1.

**Table 1.** Demographic characteristics of survey respondents (*N* = 32).

Characteristic	*n*	%
Workplace location Queensland Victoria South Australia New South Wales ACT	12 8 4 4 4	37.5 25.0 12.5 12.5 12.5
Academic level Professor A/Professor Senior Lecturer Lecturer Other	10 6 9 6 1	31.3 18.8 28.1 18.8 0.03
Role Teaching and Research Education-focused Leadership Other	22 3 3 4	68.8 9.4 9.4 12.5
Curriculum level Undergraduate Postgraduate Across courses	12 8 12	37.5 25.0 37.5
Practice background Yes No	20 12	62.5 37.5

### Sampling procedures

Participants were recruited through their educational institutions. Emails were sent to the Heads of School/Directors of Education (*N* = 48) in all Australian tertiary institutions offering accredited courses in psychology (excluding the institution of the paper authors). Each of these emails included a copy of the explanatory statement and link to the online survey. The Heads of School/Directors of Education were asked to send a copy of the statement and survey link to staff involved in curriculum development and/or delivery of teaching. Self-selection for participation was used at both the School and participant levels. The project received approval from the university human research ethics committee (project number 41843).

### Measures

The measure administered was an online survey developed by the researchers and hosted on Qualtrics XM, with data stored on secure servers. The survey included a mixture of closed- and open-ended questions collecting data to describe the respondents, how and why they included lived experience in their teaching/curriculum, as well as barriers and enablers to including this expertise (refer Appendix). The survey was designed by the researchers to ensure it was appropriate for the Australian context and, to their knowledge, there was no existing measure available.

### Procedure

The online survey was open between 22 March and 7 June 2024. After clicking on the link to the survey, participants were invited to read the explanatory statement and complete a tickbox to indicate consent. Respondents then completed the survey, which required five to ten minutes. The survey was anonymous, all questions were optional, and respondents did not receive any form of incentive or benefit for participating.

### Design

The project employed a non-experimental design for a single group. The design was grounded in a positivist approach for obtaining baseline data, as well as an interpretivist approach to understand participants’ subjective experiences and perspectives. Survey data were analysed using frequency counts and content analysis to identify recurring patterns related to the research questions. Responses were coded to themes independently by two people: the lead author and an independent research assistant. Overall, there were few differences in coding and consensus was reached through discussion of any differences.

## Results

Three broad groups of respondents could be identified from the survey: i) academics who are using and value the inclusion of lived experience in their teaching/curriculum; ii) academics who are not including lived experience and/or do not see the relevance of it for their subject areas or psychology as a discipline, and iii) academics who would like to include lived experience in their teaching/curriculum but have not had the opportunity to do so.

### Use of lived experience in psychology education

Of the 32 respondents, 56.3% reported that they include lived experience in their teaching/curriculum (see Table 2). The three most frequent approaches to this inclusion were as an example of content such as a case study, as a guest lecture, and to support development of student cultural responsiveness. The person with lived experience was typically either appointed on a sessional contract or not remunerated. No patterns were identified in the data, considering factors of whether the respondent had experience of psychology practice, taught in undergraduate or postgraduate courses, or identified as someone with personal lived experience.

**Table 2.** Inclusion of lived experience expertise in psychology curriculum (*n* = 18).

	n
Example content (e.g., case study) As a guest lecturer Support development of student cultural responsiveness Inform and/or write new curriculum Support psychosocial safety for students Co-design to inform unit learning outcomes Inform policies/as a committee member Review existing unit content As an expert response (e.g., iSAP) Support development of student skills (e.g., OSCE) Co-design to inform authentic assessment Support assessment marking	14 11 8 6 6 5 5 3 2 2 2 1
Remuneration of contributor Unpaid/voluntary Sessional academic appointment Organisational agreement Ongoing academic appointment Privately through ABN or invoice Gift card	6 5 3 2 2 2

Percentages not calculated since respondents could (and did) select multiple responses for each question. iSAP = Integrated Science and Practice. OSCE = Objective Structured Clinical Exam.

The leading reason provided as to why respondents included people with lived experience in their teaching/curriculum was to enrich learning through adding authenticity and human context (7 of 15 responses). Respondents saw lived experience inclusion as an opportunity to reflect real-world practices and make the content less abstract for students. This inclusion to enrich learning was also seen as a way to support development of competencies such as cultural responsiveness. Respondent 8 described this as:

The richness of lived and living experience and how it illuminates theoretical and clinical issues relevant to psychology practice. It provides a real and rich human context for academic learning.

The second key theme was inclusion as an ethical principle (*n* = 4), which reflected the importance of not speaking on behalf of minority groups (“*nothing about us without us*,” respondent 3), and positioning psychology within an understanding of critical theory and human rights. The third most common reason was student safety (*n* = 2), which acknowledged that students themselves often have lived experience in the context of their studies. Respondent 25 illustrates this theme with the following quote:

In psychology I believe that lived experience is a vital way we can encourage students to feel comfortable in sharing feelings and emotions. It also helps students who also have lived experience feel included and know that they can be psychologists/academics if they have experienced negative mental health.

When asked more broadly about the benefits of lived experience inclusion, regardless of whether one did or did not currently include lived experience in teaching/curriculum, similar themes emerged. Of the 17 responses, nine indicated enriching learning as the main benefit. This was often also related to building students’ personal or professional competencies. For example, respondent 10 noted:

“It will be providing students with a more authentic learning experience and one that will better equip them with an understanding of their own biases/worldview/and in many cases, privilege, from early on in their degree (if embedded right from undergraduate).

Another respondent said, “*It is an evidence based way to provide learning experiences in cultural sensitivity and to reduce stigma*” (respondent 24). The second most common theme to emerge as a benefit was student-centred learning (*n* = 4). This theme captured student satisfaction (e.g., “*When we do this, students constantly tell us it was the most profound and enjoyable part of their learning*” [respondent 28]) and student safety (“*It would help students being affected by their own LLE to share and feel safe*” [respondent 31]). The third most common benefit identified related to inclusion as a way to address systemic issues (*n* = 2), such as captured by the response that lived experience inclusion was an act of “*de-centering the ‘expert’ and dismantling power structures that contribute to epistemic and systemic injustices*” (respondent 6). Three respondents stated they did not see any benefits to lived experience inclusion and the remainder did not respond to the question.

### Barriers and enablers to Inclusion

Psychology educators were asked about their perceived barriers to including lived experience in teaching/curriculum. The most reported issue was resourcing constraints, with 55.6% of responses indicating time, funding, organisational support, and access to people were seen as key barriers by respondents. The second most endorsed theme was relevance, with eight of 27 responses indicating that the academic did not see lived experience as relevant to their teaching/curriculum (attributed to either APAC requirements or perceived irrelevance). Concerns about working with people who have lived experience were also expressed by eight respondents. There were two sub-themes evident: concern about the potential challenges of working with people who have lived experience (e.g., respondent 4 spoke of perceptions about potential “*unreliability of the people involved*”) and concern about psychosocial safety, for either the lived experience contributor and/or students. As captured by one educator:

I feel some people use the umbrella of lived experience to ‘trauma-dump’ and/or to shock others, which does not really have an educative effect. Also, trauma is highest in disadvantaged groups, and psychology academia is voyeuristic and classist (especially colleagues, if not all students). I’d be concerned that disadvantaged groups presenting their experiences would be considered specimens rather than people (respondent 27).

Seven educators provided detailed responses which spoke to tensions and concerns about lived experience inclusion in the discipline of psychology. This theme encapsulated a number of complex considerations. It was argued that lived experience inclusion is not epistemically supported, does not align with empiricism and positivism, and does not provide objective evidence-based knowledge. There was a sense of concern about political and ideological influences, as illustrated by respondent 9 who stated: “*Including LLE [lived/living experience] opens another way for educators to push particular ideological agendas onto students. Namely, they just have to choose the person with LLE expertise that will push whatever agenda they want*.” Issues such as diversity and representativeness of experience, the tension between what expertise is and how this can be contested, and ethical/practice guidelines were also raised.

Finally, psychology educators reported on what would enable them to include lived experience in teaching/curriculum. Consistent with the barriers identified, responses were a mix of practical and attitudinal. Seven of 18 responses indicated that resourcing was key to including lived experience, for example through the provision of time, budget, and access to a community network of suitable lived experience experts. Relatedly, educators felt that leadership support at a School/University executive level was important. Increased acceptability of lived experience was a theme endorsed by six respondents as an enabler for inclusion. For some academics, this was related to the acceptability of lived experience itself (e.g., respondent 3: “*More people talking about their lived experiences will bring it into discourse*”), while for others, the change was more about shifting epistemologies in psychology education (e.g., respondent 25: “*Inclusivity and acceptance of sharing life experiences in the classroom setting as a teaching tool*”).

### Educators’ personal experience

In addition to asking about lived experience inclusion in teaching/curriculum, respondents were invited to disclose if they identified as someone with psychology-related lived and/or living experience. Of the 22 respondents who self-identified as having their own personal lived experience, nine brought their personal experience to their teaching/curriculum. Reasons for doing so included relevance to the content they were teaching, a desire to reduce stigma and normalise lived experiences, modelling safe and purposeful lived experience practice (e.g., disclosure), and to support relational learning. Conversely, respondents indicated they would not include their own lived experience in their teaching/curriculum for two main reasons: (i) personal and professional boundaries, and (ii) concern about stigma and how the disclosure may be received by fellow staff, students, and the lived experience community.

## Discussion

The inclusion of lived experience is an increasing focus in health profession education, and our preliminary survey sought to provide a snapshot of the current practices within tertiary psychology education. The study found that lived experience is being used in accredited psychology courses in Australia, with the most commonly reported involvement being example content (e.g., case study), delivery of guest lectures, and to support development of cultural responsiveness. The most common reasons for inclusion were to enrich learning through adding authenticity and human context, inclusion as an ethical principle, and to support safety of students who may themselves have lived experience. Enriching learning, student-centred learning, and addressing systemic issues were identified as key potential benefits for lived experience inclusion, regardless of whether respondents already included people with lived experience in their teaching/curriculum. Resourcing, relevance, and work-safety concerns about inclusion of people with lived experience were identified as key barriers. Enablers included resourcing, leadership support, and increasing acceptance around lived experience.

This is the first study of this kind in Australia and one of relatively few studies worldwide about lived experience inclusion in tertiary psychology education. The results of this small scoping survey show that lived experience is being included in the tertiary education of psychology students in Australia and there is a group of academic staff who are committed to bringing this expertise into teaching/curriculum, or who would value doing so but have not yet had the opportunity due to systemic and perceived barriers. We also identified another group of respondents who do not use lived experience in their teaching/curriculum and do not see the relevance of it to their subject area or to the scientific/positivist epistemology that underpins psychology. Identifying these groups can assist in developing effective strategies to engage broadly with psychology academics to explore the possible value of including lived experience expertise across subject areas.

The reasons for including lived experience reported in the current study were consistent with existing health professions education research. Inclusion of lived experience can have profound impacts on both students and educators, and has been demonstrated to promote authentic connectedness between theory and real-world practice (e.g., Goodhew et al., 2022). Horgan et al. (2018) in their study of nursing education found it was important for students to interact with people with lived experience, and that student self-reflection about these interactions added value to learning. The competencies underlying psychology education in Australia aim to develop graduates who are person-centred, reflexive, culturally responsive, and able to work with sensitivity and respect for the diversity of colleagues and others they encounter in their work. It can be argued that to truly achieve this, we need to cultivate co-productive partnerships between educators, learners, and the systems and communities in which they will engage (Englander et al., 2020). Doing so does not diminish academic expertise as critical content experts, but rather immerses learners during their education in the collaborative, self-aware, and inclusive ways that we intend them to replicate in their professional work (Brand et al., 2021).

The inclusion of people with lived experience in psychology education, both as an ethical principle and a means of redressing systemic inequities, has also been highlighted in education research across other health professions. Breaking down inadvertent perpetuation of stereotypes and “othering” aligns with World Health Organization (2022) acknowledgement that lived experience inclusion helps to ensure respect for the human rights of those impacted. It is of interest to note that although 71% of respondents self-identified as having their own lived experience relevant to psychology, less than half of these respondents felt comfortable to disclose that experience in the workplace or include it in their teaching. The main reasons for not disclosing their own lived experience related to maintaining boundaries and concerns about judgement. This finding, along with the philosophical tensions reported more broadly in the study, suggests that achieving a cultural shift in the discipline towards openness and respect for lived experience will be essential for addressing barriers to lived experience inclusion.

As had been found in studies in other health professions education, several respondents indicated that lived experience is not relevant to, and/or of benefit in, the teaching of their subject area. This may be, in part, related to psychology education operating at the intersection of a scientific discipline and a professional practice, with a scope that extends beyond mental health to the science of human behaviour. Further, for some educators, knowledge is seen as being derived purely through objective science, with expertise gained through lived experience considered incompatible with this approach (Gee et al., 2016; Scholz et al., 2018). Associated with this is often a concern that the involvement of people with lived experience typically involves one person which is not representative of others (Happell et al., 2022). This is an interesting juxtaposition, given the need to train psychology graduates who have the awareness and skills to adapt their learning in order to meet the lived experiences and unique needs of the individual with whom they are working.

The barriers identified to the inclusion of lived experience in Australian tertiary psychology teaching/curriculum were also similar to those found in studies of other health professions education. The key barrier reported by respondents in the current study was resource constraints, including funding and time. This is a consistent issue that is raised across disciplines in relation to the inclusion of lived experience expertise, which has been addressed in other disciplines through measures which support sustainable involvement such as longitudinal institutional incorporation, recruitment and training of individuals who maintain engagement across courses, and clear commitment by faculty including resource and workload support (Dijk et al., 2020; Fossey et al., 2024). Such measures also strengthen organisational readiness, mitigating some of the work-safety concerns typically raised.

A second barrier reported in the current study was that the use of lived experience in the subject area was not relevant and/or did not align with the curriculum. This is a barrier that may be particularly likely to arise in psychology where curriculum is driven by accreditation requirements, with professional training commencing at postgraduate level. It may not be readily apparent that the involvement of lived experience is relevant to all curriculum areas including at foundational and pre-professional levels of education, however the experience of the authors is that this is possible, having led multiple community-involved co-design projects in our institution including in more theoretical units such as research methodology and introduction to psychology. Further, studies of undergraduate medical education highlight that incorporating lived experience expertise can address broader learning objectives applicable across curriculum, such as developing competencies in professionalism, communication, collaboration, cultural humility, and advocacy (for example, see review by Dijk et al., 2020).

Several respondents in the current study spoke of tensions and systemic issues within psychology education, such as concern about prioritising lived experience over research and practice-based knowledge. A critique often offered within our discipline is that knowledge needs to be objective and expertise gained through lived experience is seen as being incompatible with a scientific approach. However, the integration of lived experience perspectives into the scientific research process, from discovery to translation, has a long history (Beames et al., 2021), and multidisciplinary protocols for consumer and community involvement in research exist to ensure diverse perspectives can be included and represented with balance (see for example, National Health and Medical Research Council (n.d.) guidelines). It is also increasingly acknowledged that the evidence-base which has informed our ways of knowing, being, and doing in psychology reflects the time and Westernised culture in which it was developed, with growing calls for a more inclusive and critical approach to knowledge creation and dissemination that challenges traditional paradigms (Clark & Hirvonen, 2022).

Finally, it is of note that respondents indicated that lived experience inclusion was typically unpaid or remunerated sessionally rather than through an ongoing arrangement and mutual relationship. This is inconsistent with both sector guidelines (see, for example, Byrne et al., 2021; Commonwealth of Australia, 2021) and other health professions education, where ongoing positions and/or organisational partnerships which build capacity are utilised. The involvement of people with lived experience also typically occurred in an ad hoc or tokenistic manner, situated at the lower end of engagement spectrums for example, the widely used IAP2 public participation spectrum (International Association for Public Participation IAP2 International Federation, 2018). This too is inconsistent with sector guidelines and other health professions education, where more collaborative approaches such as co-design, co-production, and co-delivery are the expectation. There is a broader sense in health professions education that consumers and communities have the right to meaningfully influence the education of future professionals, shifting existing power relations prior to graduates entering the workforce (Fossey et al., 2024). We suggest that more purposeful inclusion of lived experience expertise across psychology education would model for students a health equity and human rights approach in the context of our discipline, supporting their learning towards graduate competencies.

### Strengths of this study

This is the first study, to our knowledge, on the inclusion of lived experience in accredited psychology training in tertiary institutions in Australia. It therefore provides an important initial snapshot of how people with lived experience are involved in education of our discipline nationally. This is vital information as we continue to develop psychology courses and their accreditation requirements to align with the working environments in which future graduates will be employed. Information about key barriers and enablers to the inclusion of lived experience will aid in change management and the development of frameworks and organisational practices that allow for embedding lived experience into psychology teaching and learning.

### Limitations of this study

This project was designed as a preliminary study to gain an indication of what is occurring in tertiary institutions with accredited psychology courses in Australia, and serves as a precursor to more in-depth ethnographic and participatory action research being conducted. We acknowledge that a brief survey is consistent with a positivist/post-positivist paradigm and quantitative analysis, and would have conducted more detailed analysis accordingly had response rate been higher. The open-ended questions in the survey allow for simple content analysis aligned with an interpretivist paradigm which strengthens understanding and presents multiple perspectives. It was not possible to collect information from Heads of School/Directors of Education about how many staff they invited to participate in the study, and several states did not reply to the study invitation, prohibiting calculation of response rates. We acknowledge the findings may not represent the broader tertiary psychology education community due to small sample size and self-selection bias, with 71% of respondents self-identifying as having personal lived experience. Although constrained by a small sample size and self-selection bias, the findings contribute to the limited knowledge base on the inclusion of lived experience in tertiary psychology education in Australia, providing insights into how lived experience is being integrated, as well as the barriers and enablers influencing this practice. As this work gains momentum, we anticipate an increased systematic inquiry into lived experience inclusion within the discipline nationally and overseas.

## Conclusions

Other health professions appear to be further advanced in their inclusion of lived experience in education, including several disciplines now employing lived experience designated academics or otherwise having people with lived experience inform governance and direction of curricula. Through this, they are developing an evidence base on lived experience expertise in education, along with processes to address the complexities involved. In contrast, there has been relatively little work completed on lived experience inclusion in psychology education, and our national survey of institutions resulted in limited engagement.

Recent papers suggest that psychology as a discipline in Australia, and around much of the world, has not had open discussions about the inclusion of lived experience amongst its constituents (e.g., Haywood et al., 2023; Victor, Devendorf, et al., 2022; Victor, Schleider et al., 2022). Until open discussions about lived experience and its place in psychology do occur, it is unlikely that this expertise will be meaningfully included in tertiary psychology education. As research and other health professions continue to progress in this area, psychology will inevitably face increasing pressure to align its educational practices. Finding ways to expand the epistemological landscape and share this work is crucial for our discipline, ensuring we are preparing our graduates with essential knowledge, skills, and attributes for a workforce that increasingly values consumer and community voice.

## Data Availability

Data not available. The participants of this study did not give written consent for their data to be shared publicly and due to the sensitive nature of the research and potential for identification, supporting data is not available.

## Appendix. Survey questions

1. What is your gender?
2. What is your discipline background?
3. Where is your workplace located?
4. Have you ever worked as a practising psychologist?
5. Do you identify as having a personal lived/living experience as an individual or family member/supporter of an individual, of mental ill-health or related challenges which impact mental health and wellbeing?
6. Do you disclose your personal lived/living experience expertise in the workplace?
7. Which of the following best describes your position?
8. What is your primary area of employment?
9. Are you involved in psychology teaching and/or curriculum development? What year level do you teach/develop curriculum for?
10. Do you include LLE expertise in your teaching/curriculum?
11. What motivated you to include people with LLE expertise in your teaching/curriculum?
12. How do you include LLE expertise in your teaching/curriculum? Please also provide an example where relevant.
13. When you include people with LLE expertise in your teaching/curriculum, how is this typically formalised?
14. If you identify as someone with your own LLE expertise, do you include your own LLE expertise in your teaching/curriculum?
15. What do you see as the barriers for including LLE expertise in your teaching/curriculum?
16. What do you see as the enablers for including LLE expertise in your teaching/curriculum?
17. What concerns, if any, do you have about including LLE expertise in your teaching/curriculum?
18. What benefits, if any, do you see to including LLE expertise in your teaching/curriculum?
19. Do you have anything else you would like to add about the inclusion of LLE expertise in psychology teaching and learning?
